# Decidualization of an ovarian endometrioma complicated by a sigmoid fistula during pregnancy: a case report

**DOI:** 10.1186/s13256-020-02513-7

**Published:** 2020-10-22

**Authors:** Hadiza Moutari Soule, Sofia Jayi, Tigani Guirema Madi, Alpha Boubacar Conte, Fatima Zohra Fdili Alaoui, Hikmat Chaara, Moulay Abdelilah Melhouf

**Affiliations:** Department of Gynecology - Obstetrics II, Hassan II Teaching Hospital, Sidi Mohamed Ben Abdellah University, Fez, Morocco

**Keywords:** Pregnancy, Endometrioma, Decidualization, Ostomy

## Abstract

**Background:**

During pregnancy, the discovery of adnexal masses remains frequent. Such masses are mostly benign. Ovarian endometrioma is a rare etiology. The diagnosis may be difficult in some situations, such as decidualization. It may be asymptomatic or result in complications for which magnetic resonance imaging is needed.

**Case presentation:**

We describe an unusual case of decidualization of an ovarian endometrioma complicated by a sigmoid fistula during a 7-week, 1-day pregnancy in a Arabic patient aged 38 years who developed acute pelvic pain with fever. She had a medical history of unexplored secondary dysmenorrhea. The diagnosis was suspected on the basis of magnetic resonance imaging findings. The management was based on surgery, during which exploration revealed a mass at the expense of the left ovary being very adherent and fistulized to the sigmoid. We performed adnexectomy followed by digestive ostomy. The result of pathological study with immunohistochemistry led to a diagnosis of decidualization of an ovarian endometrioma altered by infection.

**Conclusion:**

Decidualization of an ovarian endometrioma can lead sometimes to unexpected complications. The decision to provide surgery must be made with caution without delaying treatment in the event of a deep suspicion of malignancy and/or complication. The particular and exceptional complication discovered in our patient is the fistulization to the sigmoid.

## Introduction

Access to ultrasound at the beginning of pregnancy makes the association between pregnancy and adnexal mass an increasingly frequent situation [1]. Endometrioma is a rare and benign etiology of adnexal mass. Clinically, the endometrioma remains difficult to recognize because it presents with no specificity. However, its decidualization can lead to noisy complications. We report a rare case of a woman with a 7-week, 1-day pregnancy with an ovarian endometrioma decidualized and fistulized to the sigmoid.

## Case presentation

Our patient was a 38-year-old pauciparous Arabic woman with a pregnancy of 7 weeks, 1 day gestation. She had consulted for acute pelvic pain of increasing intensity with fever without metrorrhagia and any other digestive or urinary signs. She had no specific medical or family history except a notion of unexplored secondary dysmenorrhea.

On examination, the patient was hemodynamically stable and conscious, feverish at 38.6 °C, with tenderness of the left iliac fossa without defense or abdominal contracture. Her uterus was enlarged with tenderness and filling of the left lateral cul de sac.

Her biological assessment objectified hyperleukocytosis at 15,200 elements/mm^3^, hemoglobin at 11.5 g/dl, platelet count at 241,000/mm^3^, a very high C-reactive protein (CRP) at 254 mg/l, and positive leukocyturia with a negative urine culture result.

Pelvic ultrasound described an enlarged uterus that was the seat of a toned gestational sac containing an embryo of 7 weeks, 1 day with cardiac activity and yolk sac. The patient’s right ovary was without visible abnormality, and her left ovary contained two contiguous cystic images of 68 and 35 mm with thickened wall in places not vascularized on the basis of Doppler imaging, with finely echogenic content without vegetation or endocystic septum with posterior strengthening of the echoes, without effusion. We mentioned the diagnosis of hemorrhagic corpus luteum or decidualized endometrioma, given the notion of dysmenorrhea.

**Fig. 1:**
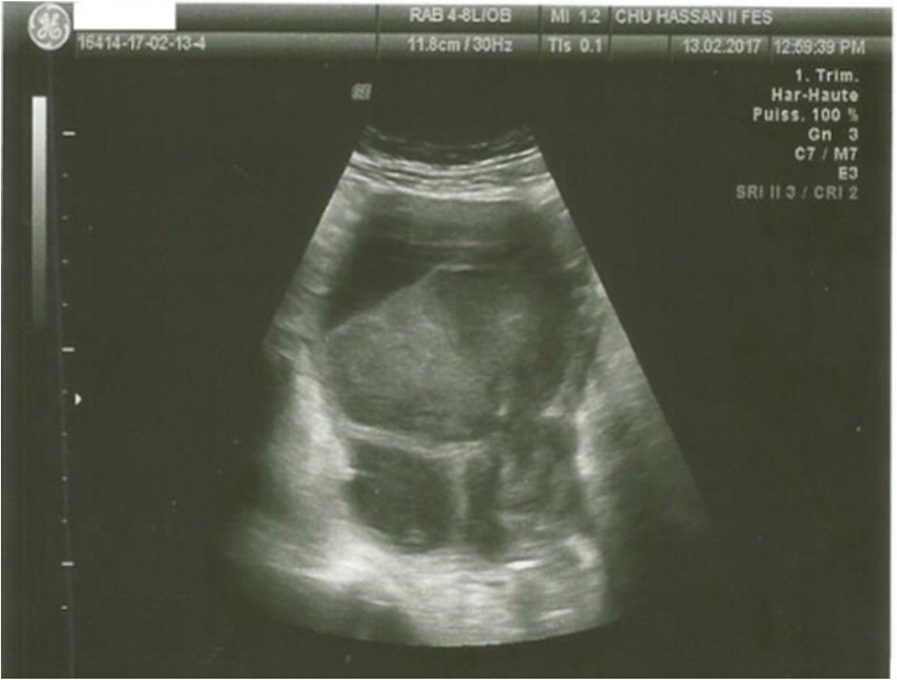
Pelvic ultrasound showing changes in cysts with a thickened wall and heterogeneous content with the appearance of a fluid level

The patient received pain relievers for her pelvic pain and antibiotics. Due to the persistence of the symptomatology (pelvic pain and fever) and the increase of CRP (294 mg/l vs 254 mg/l) after 1 week of treatment, an ultrasound control was performed, which objectified a progressive intrauterine pregnancy with persistence on the left side, of the two cysts that appeared as heterogeneous echogenic images with a thickened wall in places, pseudovegetations not taking the Doppler, and the presence of a liquid and thin effusion in the cul de sac of the pouch of Douglas (Fig. 1). The right ovary was normal.

**Fig. 2:**
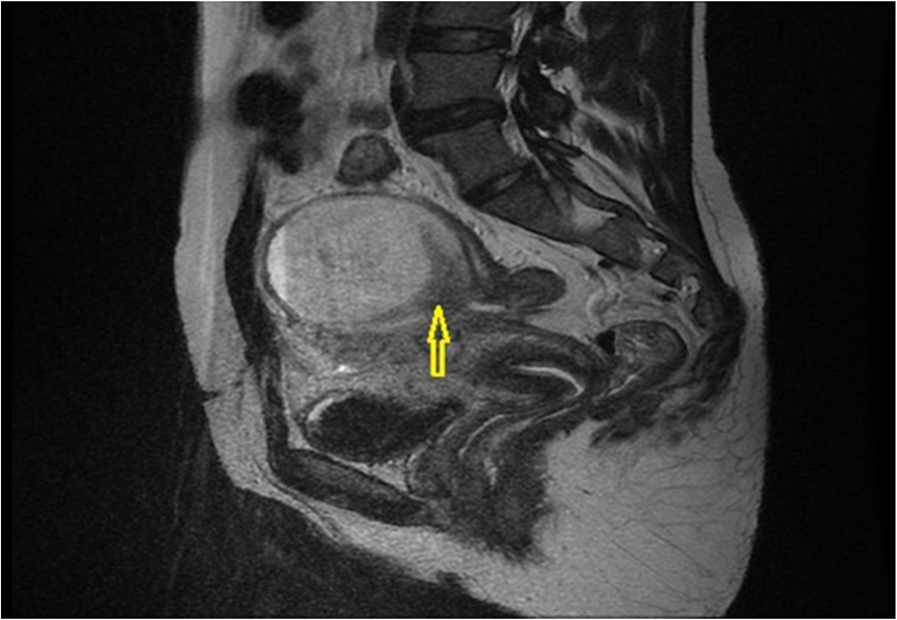
Pelvic magnetic resonance imaging in T2 sagittal section showing hypersignal of the cyst with a solution of continuity of its wall (*arrow*)

**Fig. 3:**
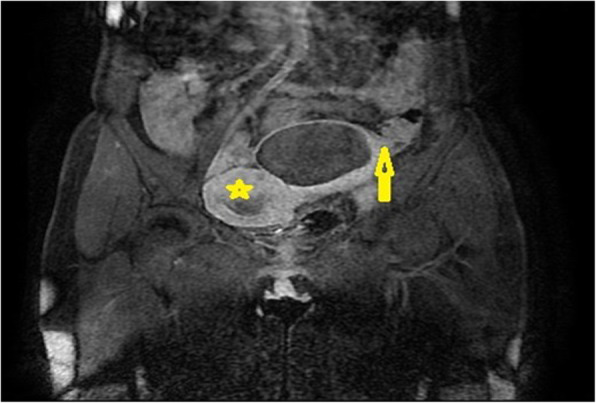
Pelvic magnetic resonance imaging in T1 coronal section after gadolinium injection showing an enhancement of the cyst wall and its close contact with the sigmoid (*arrow*) and a pregnant uterus (*star*)

Pelvic magnetic resonance imaging (MRI) was performed to complete the exploration (axial and sagittal T2 sequence, axial T1 spin echo sequence, and liver acquisition with volume acquisition before and after gadolinium injection), with the following findings: two cystic hemorrhagic formations of the left ovary in T1 and T2 hyperintensity of 87 mm × 78 mm and 25 mm × 25 mm with fluid levels without endocystic vegetation and increasing at the periphery after injection of gadolinium. The largest cyst was in intimate contact with the sigmoid with a possible rupture of its wall at this level (Figs. 2 and 3). Presence of an effusion in the cul de sac of the pouch of Douglas was detected, along with absence of infiltration of the peritoneal fat. No pelvic or lumboaortic lymphadenopathy was visible. Thus, an exploratory laparotomy was decided on the basis of radiological suspicion of sigmoid fistulization and the worsening of the patient’s clinical symptomatology.

On exploration, discovery was of a minimal serohematic effusion, a uterus at 8 weeks of gestation, and the right appendix without particularity. A mass was present at the expense of the left ovary, smooth-walled, about 10 cm and adherent to the left fallopian tube, the posterior surface of the uterus, and the sigmoid. This mass was very adherent and fistulized to the sigmoid, and there had been an accidental rupture during adhesiolysis and outpouring of a chocolate liquid. We performed a left adnexectomy and resection of the sigmoid by removing the fistula and then placing a Hartmann-type ostomy. The cytologic specimen returned in favor of an inflammatory hemorrhagic fluid without any tumor cell. Histology revealed that the cystic wall was fibrous with large foci of suppuration on the periphery. The colic wall had an ulcerated epithelium in places with broad suppurative areas at the level of the chorion. Immunohistochemistry highlighted glandular structures at the level of the intestinal wall that were well underlined by the anticytokeratin antibody and expressed estrogen and progesterone receptors with an expression of CD10 around the glands. Moreover, tumor cells were absent. Thus, the final histological study concluded the diagnosis of a decidualized and reshaped endometriotic cyst.

The postoperative follow-up and monitoring of the pregnancy were simple with full-term vaginal delivery without incident. The restoration of intestinal continuity was performed by laparotomy 6 weeks after the delivery.

## Discussion

The discovery of an adnexal mass during pregnancy poses a problem of etiological diagnosis and often leads wrongly to surgical management [1]. Only 1–8% of adnexal masses operated in pregnant women are of malignant origin [2, 3]. Ectopic deciduosis is incidental and sometimes asymptomatic. When present, the signs of ectopic deciduosis are not very specific. On the other hand, ectopic deciduosis can be revealed by a noisy symptomatology related to a complication. The interrogation allows clinicians to orient themselves by looking for a history of endometriosis or even intense or disabling dysmenorrhea. This was the case in our patient, who reported the notion of intense and chronic primary dysmenorrhea.

In ultrasound, the diagnosis of decidualization is all the more difficult because the image sometimes leads to confusion with a malignant tumor, regardless of the digestive, ovarian [4], or bladder location [5]. However, ultrasound remains the first-line examination in the setting of any adnexal mass, even during pregnancy. The endovaginal route is recommended in the first trimester, whereas the suprapubic route is preferred in the second and third trimesters [1]. The endometrioma presents itself as a homogeneous echogenic image not vascularized with Doppler imaging, a discrete posterior reinforcement, and small hyperechogenic points [6]. After decidualization, it remains with a finely echogenic, homogeneous content, with pseudovegetative structures very vascularized with Doppler, which actually corresponds to festoons of the thickened wall [1].

In our patient’s case, ultrasound had objectified two finely echogenic cystic images with a wall thickened in places but not taking the Doppler and then at the control imaging, 1 week later, the appearance of an intracystic fluid level evoking hemorrhage associated with pseudovegetations.

In patients with known endometriosis, the diagnostic orientation is quickly established. Otherwise, it is the bilaterality, the multifocality, and/or the presence of peritoneal lesions that will evoke the diagnosis [7].

However, in certain cases, in particular those with complications or doubt about the malignancy, MRI is necessary, which was the case in our patient. It has been reported that these situations must always have an MRI performed to better guide the diagnosis [1, 7].

Even if it was preferred previously from the second trimester, MRI is now authorized by some American and European authors, regardless of the patient’s gestation, if the maternal benefit is significant [8].

The signal characteristics are those of an endometrioma with vegetation-like formations on the wall in T1 hyposignal and characteristic T2 hypersignal. The gadolinium injection will not make the difference between a borderline tumor or a decidualization [1].

In our patient’s case, MRI was performed with gadolinium injection and objectified two cystic formations of the left ovary in hyperintensity T1 and T2. We did not objectify a vegetative formation on the cystic wall, but it increased after injection of gadolinium. In addition, one of the cysts was in close contact with the sigmoid with suspicion of a rupture of the wall at this level.

Macroscopically, the endometrioma contains a thick chocolate liquid with adhesions to neighboring structures [9]. Some authors report that macroscopic examination of the endometrioma has a sensitivity of 97% and a specificity of 95% [10] and a predominance of endometriomas in the left ovary [11].

In histology, although difficult to differentiate it from the hemorrhagic cyst, it is reported by many authors that the wall of the endometrioma is so laminated that it contains only a simple cubic epithelium surrounded by fibrosis [12].

In our patient, the cyst actually sat at the level of the left ovary with a macroscopic aspect of endometrioma, and the final histology of the wall as well as immunohistochemistry were compatible with what the literature reports.

We asked ourselves the question whether the ultrasound characteristics and those of MRI in our patient’s case have not been modified by alterations linked to infection. The pathologic examination answers this question by specifying the changes undergone by the cyst.

Abstention is the attitude recommended by large numbers of authors, with regular ultrasound monitoring for ectopic deciduosis and masses whose characterization on imaging was sufficient for diagnosis [13–15]. However, during monitoring, complications such as hemorrhages, pain linked to adhesions with neighboring organs, and a previa obstacle, depending on the size and the seat, may occur [16, 17] and indicate surgery.

Apart from any complications and after delivery, there is a total regression of the changes in the wall of the endometrioma, which takes the appearance of a simple endometrioma [1, 5].

## Conclusion

A decidualized ovarian endometrioma can lead sometimes to unexpected complications. The decision to perform surgery must be made with caution without delaying treatment in the event of a strong suspicion of malignancy and/or a complication. The particular and exceptional complication discovered in our patient is the fistulization to the sigmoid, which explains the persistence of the clinical symptomatology. The infection was probably a cause of the ultrasound and MRI changes. Histology was key to the diagnosis despite changes due to the infection.

## Data Availability

All the data available are presented in this report.

## Abbreviations

CRP: C-reactive protein
MRI: Magnetic resonance imaging
